# Epidemiological distribution of respiratory viral pathogens in marketable vaccinated broiler chickens in five governorates in the Nile Delta, Egypt, from January 2022 to October 2022

**DOI:** 10.14202/vetworld.2024.303-312

**Published:** 2024-02-07

**Authors:** Ahmed Ali El-Shemy, Mohamed Mahrous Amer, Heba M. Hassan, Mohamed Elaish

**Affiliations:** 1 Department of Parasitology and Animal Diseases, Veterinary Research Institute, National Research Centre, P.O. 12622, Dokki, Giza, Egypt; 2 Department of Poultry Diseases, Faculty of Veterinary Medicine, Cairo University, P.O. Code 12211, Giza, Egypt; 3 Agriculture Research Center, Animal Health Research Institute, Dokki, PO. Box 246, Giza 12618, Egypt

**Keywords:** avian influenza virus, chicken, Egypt, infectious bronchitis, Newcastle disease, reverse transcriptase polymerase chain reaction, subtype H5 and H9

## Abstract

**Background and Aim:**

Respiratory viral infections significantly negatively impact animal welfare and have significant financial implications in the poultry industry. This study aimed to determine the frequency of the most economically relevant respiratory viruses that circulated in Egyptian chicken flocks in 2022.

**Materials and Methods:**

Chickens from 359 broiler flocks in five different Egyptian governorates in the Nile Delta (Beheira, Gharbia, Giza, Monufiya, and Qalyoubia) at marketing time (33–38 days of age) were used in this study. Combined oropharyngeal and cloacal swabs and tissue samples were collected from clinically diseased or freshly dead birds suffering from respiratory disease. Avian influenza (AI)-H5, AI-H9, Newcastle disease (ND), and infectious bronchitis virus (IBV) were analyzed by reverse transcriptase polymerase chain reaction.

**Results:**

Of the 359 flocks examined, 293 tested positive, whereas 66 were completely negative for the four viruses evaluated, with the highest positive results in Beheira. Out of 293 positive flocks, 211 were positive for a single virus, with Beheira having the highest rate, followed by Qalyoubia, Giza, and Monufiya. ND virus (NDV) was found to be the highest across all governorates, followed by IBV, AI-H9, and AI-H5. A double infection was detected in 73 flocks with either H9 or ND, or both H9 and IB could coinfect each other. The most common viral coinfections were H9 + IB, ND + IB, and ND + H9. Giza had the highest prevalence of ND + H9, H9 + IB, and ND + IB coinfection in the governorates, followed by Monufiya and Beheira. Only six out of 359 flocks were tribally infected with ND + H9 + IB in Giza, Monufiya, and Beheira governorates. On the basis of the number of flocks and the month of the year, July had the lowest number of flocks (23), while September and October had the highest number (48 flocks). Positive flock numbers were highest in October and lowest in January.

**Conclusion:**

From January to October 2022, prevalent respiratory viral infections (H5N1, NDV, H9N2, and IBV) were detected in broiler chickens across the Delta area governorate, according to the findings of the present study. In addition, IBV and H9, either alone or in combination, significantly contributed to the respiratory infection observed in broiler chickens. Regardless of the type and origin of the vaccine used, it is not possible to protect broiler chickens from the development of the infection and the subsequent dissemination of the virus into the poultry environment. In the presence of face-infectious field virus mutations, poultry vaccinations must be regularly reviewed and updated, and poultry farms must take further biosecurity measures.

## Introduction

Respiratory coinfections are more common in poultry due to the presence of several causal agents. In addition to significant financial implications, respiratory viral infections in the poultry industry significantly negatively impact animal welfare. As respiratory disease in chickens clinically worsens, it becomes difficult to establish a clear diagnosis and find an appropriate therapy. Accordingly, both precipitating causes and predisposing variables should be addressed in respiratory complex infection control efforts [1, 2].

The pathogens cause respiratory diseases in poultry work independently or in combination. As a result of their multifaceted nature, respiratory diseases are a significant challenge in Egypt’s poultry sector. A number of poultry respiratory infections have similar clinical indications that can be misinterpreted. In recent years, a number of clinical indications have become more common in Egyptian commercial chicken flocks. These infections are significant and have a significant economic impact because they can cause disease individually or in combination [3]. The primary respiratory causes of high death rates in broiler chicken flocks include infectious bronchitis virus (IBV), Newcastle disease virus (NDV), and avian influenza (AI), which can be highly pathogenic (HP) as H5 or low pathogenic (LP) as H9 [4].

Infectious bronchitis (IB), a highly contagious disease that causes large economic losses to commercial chicken flocks, is one of the main contributors to mixed infections. IBV belongs to the family *Coronaviridae* and many distinct IBV variants circulate worldwide [5]. The virus usually causes mild respiratory disease, decreases egg production, and can induce renal damage depending on the viral variant [6]. Chickens are susceptible to IBV infection. Reduced weight gain, poor feed efficiency, and an increase in carcass condemnations in broilers result in economic losses, particularly when secondary infections (e.g., bacterial infections) exacerbate IB [7]. IBV frequently causes respiratory symptoms such as gasping, coughing, sneezing, tracheal rales, and nasal discharge [8], and some IBV strains have been linked with kidney lesions [9]. The symptoms of chickens vary according to age and immune status, with virus strains affecting pathogenesis and organ lesions. The primary site of infection is the upper respiratory tract, but it can also replicate in the reproductive, renal, and digestive systems [10]. Polymerase chain reaction (PCR) on reversely transcribed RNA is a potent technique for the detection of IBV to monitor different IBVs in a geographical region. PCR-based techniques are both sensitive and fast compared with classical detection methods [11].

Newcastle disease (ND) is one of the most severe viral infections in poultry, with velogenic strains of the virus causing 100% bird mortality in affected flocks [12]. NDV is considered endemic in many countries, including Egypt [13]. Exotic NDV is an HP virus that causes significant losses in domestic and game birds in the United States. It is also considered that these birds are an excellent reservoir for the transmission of the virus between domestic commercial flocks, leading to significant losses in the poultry sector [13]. NDV infection can be classified into pathotypes according to the strain infecting the flock and the clinical symptoms it causes. There are four distinct strains. Lentogenic strains cause mild respiratory symptoms and are used as secondary live vaccines. Mesogenic strains kill only young chicks. Viscerotropic velogenic strains kill chickens of all ages and usually appear as enteric signs. Neurotropic velogenic strains kill chickens of all ages and usually manifest as nervous signs [14, 15]. In recent years, the mutations and genetic diversity of NDV have increased [16–19]. The wide genetic distance between the currently circulating NDV isolates and the vaccines used to combat the disease is an area of debate in many countries [18, 20].

AI is a poultry disease that causes significant economic losses in addition to its zoonotic potential. Orthomyxoviridae Type A influenza viruses are responsible for this disease. AI is further divided into HPAI and LPAI virus strains according to the severity of the disease in vulnerable birds. The H5 and H7 subtypes are mostly responsible for HPAI outbreaks in hens and turkeys; however, some strains of these subtypes are known to be LPAI [21]. The slow spread of LP H9N2 in the Middle East and the Far East has been identified as another risk for the poultry industry. H9N2 viruses are highly lethal [22]. Although classified as LPAI viruses, H9N2 virus emergence from a clinically healthy commercial bobwhite quail flock in Egypt occurred in May 2011, and the virus cocirculated with HPAI virus (HPAIV) subtype H5N1, with the potential for H9N2 virus quiet spread to disrupt the normal spread of HP H5N1 [23, 24].

AI virus (AIV) and IBV are the two main viruses that infect chicken airways. LPAI causes mild respiratory symptoms, egg production loss, and growth reduction [25, 26]. Dual infections with two viruses or even more infectious agents are more common in chickens [27]. However, infection with avian respiratory viruses (IB, AI, NDV, or avian metapneumoviruses) increases the susceptibility to subsequent respiratory bacterial infections [25, 28, 29]. Furthermore, immunosuppressive viruses, such as chicken anemia virus, can dramatically increase the risk of subsequent infections [30]. The ensuing disease is typically more severe when two pathogens coexist with each other [31, 32]. Despite numerous proposed underlying mechanisms of virus–virus interactions, disease mechanisms underlying infection with two viruses have only occasionally been examined in the context of chicken respiratory disease [27, 33].

In Egypt, commercial chicken flocks have experienced ongoing outbreaks of respiratory diseases with varying mortality rates and clinical manifestations [34]. According to recent research, the most prevalent condition in Egyptian poultry is mixed infection, especially with IB and H9N2 viruses [29]. A recent *in vivo* study found that dual infection with the first IBV and the second AIV causes a more severe clinical illness with an increased inflammatory response, but comparisons are still difficult because no sequential IBV infections or receptor binding have been studied [35]. A mechanistic study revealed that diverse levels of epithelial susceptibility to secondary viral infections are associated with the loss of cilia and goblet cells in IB-induced tracheitis [33]. Binding reduction of the two IBV variants was more prevalent than that of IBV and AIV. Superinfection reduced viral titers *in vivo*, *in ovo*, and *in vitro* but did not affect the replication of the second virus. Reducing surface receptor molecules is the main antiviral mechanism, but it works differently for each virus. It may be controlled by interactions between cytokines and interferons or sialidase activity in the host [36].

The degree of binding reduction differed according to virus and was more common between the two IBV variants than between IBV and AIV. Superinfection lowered the viral titers of the first injected virus *in vivo*, *in ovo*, and *in vitro* (cell cultures); however, it had no effect on the replication of the second virus, regardless of which virus was inoculated first or second. An underlying epithelial antiviral mechanism-based surface receptor molecule reduction that varies for different viruses could be regulated by an interaction between different cytokine and interferon types or host-induced sialidase activity rather than a virus-specific mechanism primarily based on competition for the same susceptible cells, as concluded in one of the previous studies by Kong *et al*. [35].

The study was conducted for molecular identification of the prevalence of the most economically relevant respiratory viruses circulating in chicken flocks in Egypt throughout 2022 due to the ongoing mutation of AI, ND, and IBVs and the record of their breakouts in vaccinated chickens.

## Materials and Methods

### Ethical approval

The National Research Center’s Medical Research Ethics Committee (Decision No. 20234) granted ethical approval for this study. The study involved swabs and tissue samples collected from Egyptian poultry farms by poultry veterinarians. The samples collected in this study, transportation, and processing were performed in compliance with the National Research Center’s Medical Research Ethics Committee.

### Study period and location

This study was conducted from January 1 to October 31, 2022. Samples were collected from five different Egyptian governorates in the Nile Delta, where poultry farms are concentrated. The swabs collected from each poultry farm were pooled and represented as one sample. Beheira was the governorate (sample No. = 201), Gharbia (sample No. = 23), Giza (sample No. = 28), Monufiya (sample No. = 89), and Qalyoubia (sample No. = 18), as summarized in Table-1. For further analysis, samples were submitted to the National Research Center, Faculty of Veterinary Medicine, Cairo University and the Egyptian Laboratory for Poultry Health for further analysis.

**Table-1:** Number and percentage of RT-PCR positive and negative broiler flocks about the governorate.

Governorate	No. of tested flocks	Positive flocks		Negative flocks	
	
		No.	%	No.	%
Beheira	201	169	84.1	32	15.9
Gharbia	23	18	78.3	5	21.7
Giza	28	22	78.6	6	21.4
Monufiya	89	71	79.8	18	20.2
Qalyoubia	18	14	77.8	4	22.2
Total	359	293	81.6	66	18.4

RT-PCR=Reverse transcriptase polymerase chain reaction

### Chicken flocks

The flocks were raised on floor-open farms with capacities ranging from 3000 to 50,000 birds. Commercial ready-made pelted rations are used according to the standard requirements of each breed. Birds were vaccinated against H5 (inactivated or vector vaccine), H9 (inactivated), ND (live, inactivated, or vector) (Vaxxtek, Georgia, USA), and IBV (live against classical or variant strains).

### Sampling

Combined oropharyngeal and cloacal swabs and tissue samples were collected from clinically diseased or freshly dead birds (3–5 samples/flock) suffering from respiratory disease problems at the flock marketing time (33–38 days of age). The samples were transported to our laboratory, where the liver, lung, spleen, and trachea were pooled as one sample, tagged, and stored in a sterile plastic bag at −20°C. Tissue samples were ground in 1:5 (w/v) phosphate-buffered saline (PBS) with gentamicin (50 g/mL) and myostatin (1000 units/mL) at pH 7.0–7.4, centrifuged, and the tissue supernatant recovered. Swabs from each flock were combined and suspended in 2 mL of PBS, pH 7.0–7.4, cleared by centrifugation at 1740× *g* for 10 min, and then processed for RNA extraction [37, 38].

The current investigation was conducted in five governorates of the Nile Delta, which have the greatest incidence of outbreaks and the highest occurrence of diseases due to the dense presence of poultry and human activities. In a previous study by Yehia *et al.*, [39] the prevalence of AI, IB, and ND were 39.6%, 37.7%, and 5.7%, respectively. To calculate the total sample size with 5% accuracy and a 95% confidence level, the prevalence of ND (5.7%) was chosen. We calculated the number of sampled farms and provinces using the formula cited by Haji-Abdolvahab *et al*. [28]:

### Real-time PCR

#### Extraction of viral RNA

The QIAamp Viral RNA Mini Kit (Qiagen, Valencia, Calif., USA, Cat. no. 52904) was used to extract the RNA according to the manufacturer’s recommendation. Different causative agents of these diseases were investigated using real-time quantitative reverse transcriptase PCR (RT-PCR) and the QuantiTect probe RT-PCR kit (Qiagen, Inc., Cat no. 204443).

#### Primer and probe used for RT-PCR

Primers and probes used for RT-PCR were designed as shown in Table-2 [40–43]. In brief, primers for AI-H5, AI-H9, ND, and IBV were designed according to previous studies [40–43].

**Table-2:** RT-PCR primers against tested viruses.

Primer	Type	Primer sequence (5′-3′)	Reference
H5N1	F	5-ACATATGACTAC CCACARTATTCA G-3	[41]
	R	5-AGACCAGCT AYC ATGATTGC-3	
	Probe	5-FAM-TCWACA GTGGCGAGT TCCCTAGCA-TAMRA-3	
H9	F	5-GGA AGA ATT AAT TAT TAT TGG TCG GTA C-3	[42]
	R	5-GCC ACC TTT TTC AGT CTG ACA TT-3	
	Probe	5-FAM- AAC CAG GCC AGA CAT TGC GAG TAA GATCC –Tamra-3	
IB	F	5-GCT TTT GAGCCT AGC GTT-3	[40]
	R	5-GCC ATG TTG TCA CTG TCT ATT G-3	
	Probe	5-FAM-CAC CAC CAG AAC CTG TCA CCT C-BHQ1-3	
ND	F	5′-GGTGAGTCTATTCGGARGATACAAG-3′	[43]
	R	5′-AGCTGTTGCAACCCCAAG -3′	
	P	5-[FAM] AAGCGTTTCTGTCTCCTTCCTCCA[TAMRA]-3	

RT-PCR=Reverse transcriptase polymerase chain reaction, ND=Newcastle disease, IB=Infectious bronchitis

## Results

**Figure-1:**
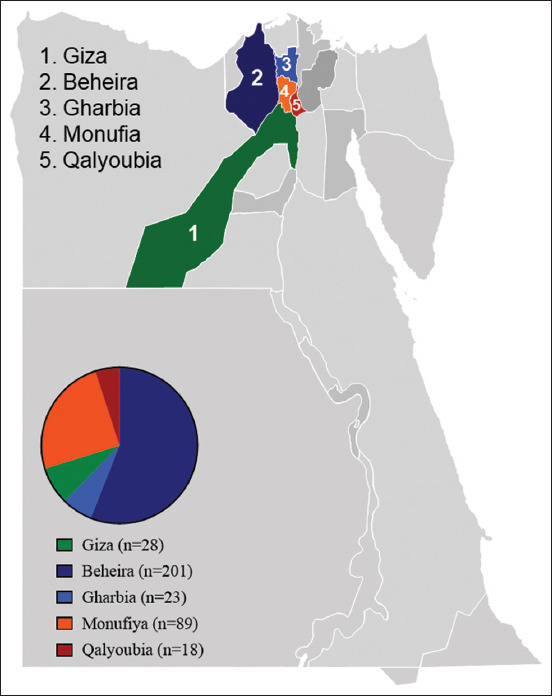
Geographic distribution and cumulative number of poultry sampled in different governorates of the Delta region in Egypt during 2022. Pie charts depict the number of samples collected from each governorate [Source: Map was adapted from PresentationGO.com – The free PowerPoint and Google Slides template library].

Three hundred and fifty-nine flocks of broiler chickens from five different governorates with respiratory issues were evaluated (Table-1 and Figure-1). Different clinical symptoms and post-mortem lesions were observed in the respiratory disease outbreaks, depending on the virus strain that caused the epidemic, how often the flock was vaccinated, and whether the illness was caused by a single infection or several infections. The primary clinical signs of respiratory distress were gasping, rales, and nasal secretions. There have been reports of head tilting in some flocks as a result of nervous disturbance. During postmortem examination, tracheitis, tracheal caseation at the tracheal bifurcation, and engorged viscera were the main pathological findings. In addition, pneumonia, enlarged kidneys, and thickening and cloudy air sacs were observed. In addition to the respiratory system, lesions were observed in the central nervous system, the gastrointestinal tract, and tissues, including the kidney.

**Figure-2:**
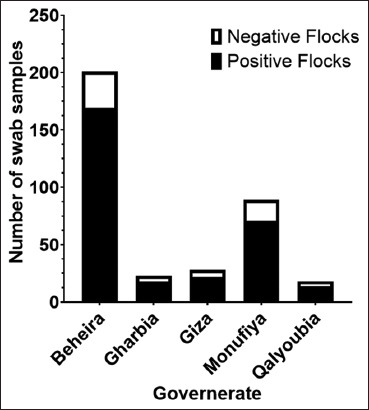
Overall frequency of positive and negative samples evaluated across five different Egyptian governorates during 2022.

Out of the 359 flocks that were tested, swab samples showed that 293 (81.6%) were positive for one of the pathogenic respiratory viruses, while only 66 (18.4%) were negative for all four viruses (Table-1 and Figure-2). The highest positive results were recorded at 84.1% in Beheira, followed by 79.8%, 78.6%, 78.3%, and 72.2% in Monufiya, Gharbia, Giza, and Qalyoubia, respectively.

**Figure-3:**
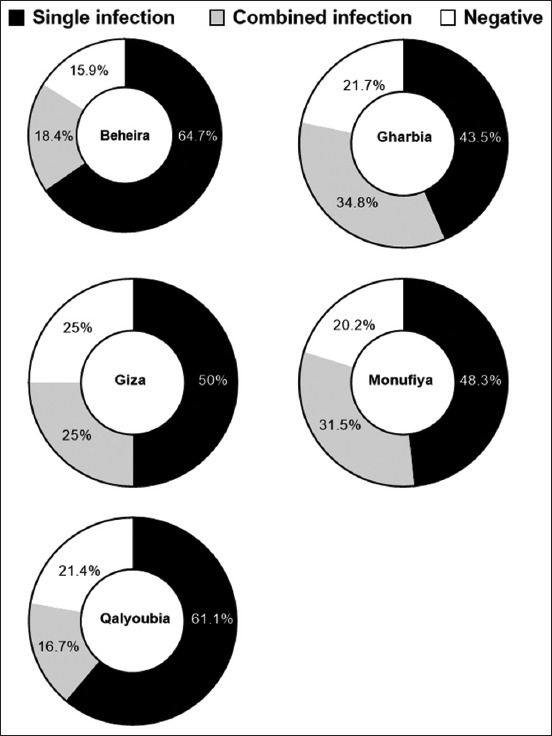
Summary of different infections (single, combined, and negative) of H5, H9, Newcastle disease virus, and infectious bronchitis virus in each governorate.

**Figure-4:**
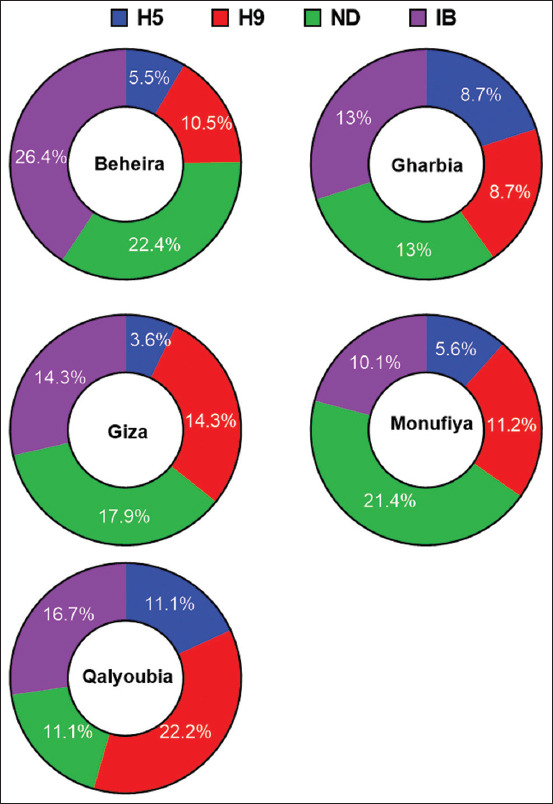
Single infections pie charts of H5, H9, Newcastle disease virus, and infectious bronchitis virus in each governorate.

Tested flocks showed that 211 (58.7%) were positive for a single virus, with the highest rate observed in Beheira (64.7%), followed by Qalyoubia (61.1%), Giza (50.0%), and Monufiya (48.31%) (Table-3 and Figure-3). NDV was the highest detectable virus in all governorates (11.1%–22.4%), with a total rate of 20.6%. IBV was the second most prevalent (10.1%–26.4%), with a total rate of 20.1%. AI-H9 was the third most common in the range of 8.7%–22.2%, with a total rate of 11.4%. AI-H5 was the least detectable pathogen in our tested samples (3.57%–11.11% and 5.85%, respectively, Table-3 and Figure-4).

**Table-3:** Number and percentage of RT-PCR-positive broiler flocks for single infection about the governorate.

Governorate	No. of tested flocks	H5		H9		ND		IB		Total single infection	
				
		No.	%	No.	%	No.	%	No.	%	No.	%
Beheira	201	11	5.5	21	10.5	45	22.4	53	26.4	130	64.7
Gharbia	23	2	8.7	2	8.7	3	13.0	3	13.0	10	43.5
Giza	28	1	3.6	4	14.3	5	17.9	4	14.3	14	50.0
Monufiya	89	5	5.6	10	11.2	19	21.4	9	10.1	43	48.3
Qalyoubia	18	2	11.1	4	22.2	2	11.1	3	16.7	11	61.1
Total	359	21	5.9	41	11.4	74	20.6	72	20.1	211	58.8

RT-PCR=Reverse transcriptase polymerase chain reaction, ND=Newcastle disease, IB=Infectious bronchitis

**Figure-5:**
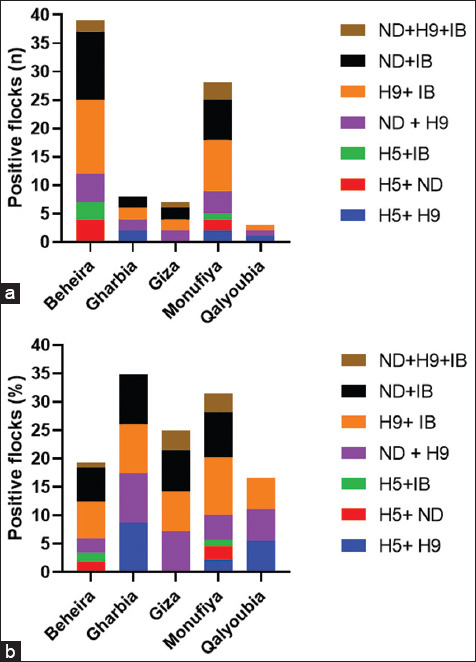
Overall frequency of double and triple respiratory infection detected in the affected flocks. The frequencies were calculated relative to the total number of isolates (n = 359). The data were presented as numbers in each governorate (a) and percentage (b).

**Figure-6:**
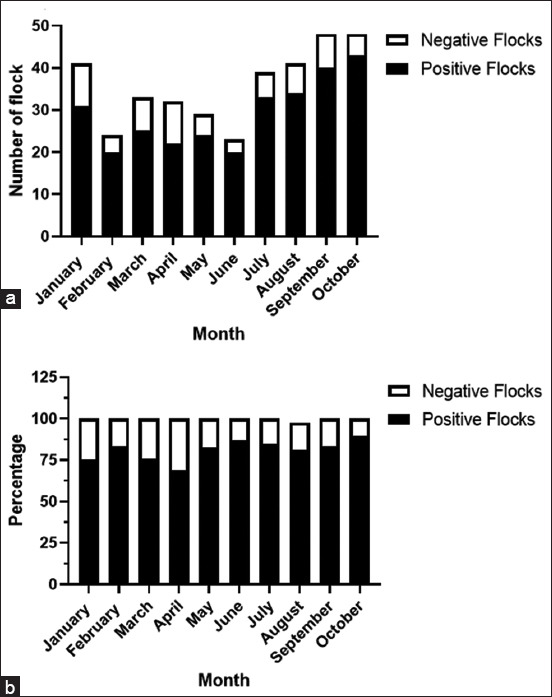
Seasonal distribution of positive and negative samples in the affected flocks. The data were presented as numbers in each month in all examined governorates (a) and percentage (b).

In 73 flocks, there was a double infection, with an average rate of 20.33%. The highest rate of co-infection between H9 + IB (7.52%), ND + IB (6.41%), and ND + H9 (3.90%) was observed, whereas 1.67%, 1.39%, and 1.11%, respectively, shared ND, H9, and IB, respectively. Regarding coinfection with ND + H9, H9+ IB, and ND + IB in the governorates, Giza had the highest distribution, followed by Monufiya and Beheira. Both H9 and IB mostly coinfected each other and either H9 or ND (Figure-4). Only six of the 359 flocks (1.7%) had ND + H9 + IB tribal infection in three governorates: Giza (3.57%), Monufiya (3.37%), and Beheira (0.9%) (Figure-5 and Table-4). The number of flocks per year was the lowest in Julie (23 flocks), and the highest in both September and October (48 flocks). Positive flocks had the highest rate in October (89.58%), followed by June (86.66%), July (84.62%), and January (75.61%) (Table-5 and Figure-6).

**Table-4:** Number and percentage of RT-PCR-positive broiler flocks for double and triple infection about the governorate.

Governorate	No of flocks	H5 + H9		H5 + ND		H5 + IB		ND + H9		H9 + IB		ND + IB		Total		ND + H9 + IB	
							
		No.	%	No	%	No	%	No	%	No	%	No.	%	No.	%	No	%
Beheira	201	0	0	4	1.9	3	1.5	5	2.5	13	6.5	12	5.9	35	17.4	2	0.9
Gharbia	23	2	8.7	0	0	0	0	2	8.7	2	8.7	2	8.7	8	34.8	0	0
Giza	28	0.0	0.0	0	0	0	0	2	7.1	2	7.1	2	7.1	6	21.4	1	3.6
Monufiya	89	2	2.3	2	2.3	1	1.1	4	4.5	9	10.1	7	7.9	25	24.7	3	3.6
Qalyoubia	18	1	5.6	0	0	0	0	1	5.6	1	5.6	0	0	3	16.7	0	0
Total	359	5	1.4	6	1.7	4	1.1	14	3.9	27	7.5	23	6.4	73	20.3	6	1.7

RT-PCR=Reverse transcriptase polymerase chain reaction, ND=Newcastle disease, IB=Infectious bronchitis

**Table-5:** Number and percentage of RT-PCR-positive and negative broiler flocks with sampling month.

Month	No. of evaluated flocks	Positive		Negative	
	
		No	%	No	%
January	41	31	75.6	10	24.4
February	24	20	83.3	4	16.7
March	33	25	75.8	8	24.2
April	32	22	68.8	10	31.2
May	29	24	82.8	5	17.2
June	23	20	87.0	3	13.0
Julie	39	33	84.6	6	15.4
August	41	34	82.9	7	17.1
September	48	40	83.3	8	16.7
October	48	43	89.6	5	10.4
Total	359	293	81.6	66	18.4

RT-PCR=Reverse transcriptase polymerase chain reaction

## Discussion

As a result of the rapid expansion of Egypt’s poultry farming industry, as well as the global transport and trade of poultry, numerous viral diseases have emerged and spread [44]. This study estimated the prevalence of respiratory viral pathogens such as AIV subtypes H5 and H9, IBV, and NDV in broiler chickens. From an economic point of view, these are some of the most important diseases for Egypt’s poultry industry.

The clinical signs findings of this study are consistent with those reported by previous studies [45, 46] that the primary clinical signs of respiratory disease included nasal and ocular discharge, dyspnea, gasping or open mouth breathing, and mortality in a flock. Occasionally, nervous signs were also observed [45, 46].

Pneumonia, enlarged kidneys, and cloudy and thickened air sacs were also observed. In addition to the respiratory system, lesions were observed in the gastrointestinal tract, central nervous system, and tissues such as the kidney. These results are in accordance with previously published studies in Egyptian flocks [34, 45, 46].

Of the 359 flocks evaluated, 293 (81.6%) were positive for common respiratory virus infection (H5N1, H9N2, NDV, and IBV), whereas only 66 (18.4%) tested negative. Behira had the largest percentage (84.08%), followed by Monufiya, Gharbia, Giza, and Qalyoubia with 79.8%, 78.8%, 78.6%, and 72.2%, respectively (Table-1 and Figure-2). Mixed infection with IB and AIV-H9 was observed in 66.3% (57 flocks), whereas a single viral infection of the tested viruses was observed in 33.7% (29 flocks) [34].

This study detected multiple respiratory viruses as single or mixed infections. NDV was detected at the highest percentage (11.1%–22.4%), with a total rate of 20.6%. With a total rate of 20.1%, IBV ranks second (10.1%–24.4%). H9N2 ranked third in the range (8.70%–22.2%) with a rate of 11.42% The H5N1 virus has a lower range of 3.57%–11.11% and a total rate of 5.85% (Table-3 and Figure-3). In Egypt, the most common cause of respiratory affection was mixed infection with IB and AIV-H9 viruses, which was the most common situation in the examined flocks [34]. A recent *in vivo* study found that dual infection with the first IBV and the second AIV causes a more severe clinical illness with an increased inflammatory response, but comparisons remain difficult because no sequential IBV infections or receptor binding have been studied [47]. The mechanism behind this finding is that the loss of cilia and goblet cells in IBV-induced tracheitis is associated with varying epithelial susceptibility to secondary viral infections [33]. Binding decreases in the IBV and AIV types differed depending on the virus. Superinfection lowered viral titers *in vivo*, *in ovo*, and *in vitro*. However, there was no effect on the replication of the second virus, irrespective of the initial and second inoculated viruses. These observations suggest an underlying epithelial antiviral mechanism-based surface receptor molecule reduction that varies among different viruses. This mechanism may be regulated by interactions between different cytokines and interferon types or host sialidase activity [36].

AIV (HP, LP), IBV, and NDV were the main viruses detected alone or in combination in broiler chickens [34]. In recent years, the AI situation in Egypt has become more complicated due to the detection and circulation of many serotypes, including HPAI (H5N1), HPAI (H5N8), and LPAI (H9N2) [48, 49]. Yehia *et al*. [39] reported that AIV subtypes and IBV were recorded in 48 out of 53 farms. A single infection accounted for 90.6% (37.7% I.B., 30.2% H5N8, 9.4% I.B., and 5.7% NDV) (Table-4) The copresence of HPAI (H5N8) and IBV, the unique detection of these coinfected flocks, and LPAI (H9N2) and IBV were detected in 3.8% flocks, as previously reported by El-Shall *et al*. [46]. Another study [29] reported that 35, 27, 12, 9, and 9 samples were positive for HPAIV H5N8, H9N2, IBV, HPAIV H5N1, and NDV, respectively, out of the 39 farms investigated. In the majority of farms, different combinations of these viruses were detected, whereas single infections were detected in only 7 of 39 (17.9%) of the farms. Cocirculation of HPAIV H5N8 and H9N2 was the most commonly detected mixed infection (11/39 farms, 31.8%).

In the present study, 1.4% circulation of the H5 and H9 subtypes of AIV (5/359) could be a risk for the emergence of new AI viruses (Table-4 and Figures-3 and-4), which complies with previous results by Gado *et al*. [49]. Forty-two layer and broiler chicken farms (21 each) suspected of different breeds and ages being infected with AIV were examined at Sharkia Governorate during 2012–2014. Using the HI assay, the incidence of AI in chickens was 40.47%. The H9 and H5 subtypes were subtyped by specific antisera. H9 and H5 were detected in 30.95% and 9.52%, respectively [50–52]. Under field conditions, AI-H9 coinfection increased the severity of other pathogens [25], including IB [4, 31, 35, 47] or H5 [49, 53, 54] or ND [55, 56], as well as the severity of *Escherichia coli* [57] and secondary bacterial infection [58, 59].

Egypt uses a variety of commercial AIV vaccines to combat H5 infections and ongoing deaths in flocks that received vaccinations due to genetic differences between previously identified Egyptian HPAIV and recently mutated strains [29, 51, 60–62]. In addition, experimental evidence has revealed the emergence of different mutants even within the same host during infection [63] or within single vaccine lots [64]. In addition, vaccine strains H120 and 4/91 were not able to provide effective protection against virulent IBV [65]. The available IBV vaccines do not provide effective cross-protection against strains of different serotypes [66]. It has also been reported that NDV avirulent and virulent strains can be isolated from chickens, regardless of their origin or vaccination status [18, 67]. Improper vaccination may be involved in ND outbreaks in vaccinated chickens [18, 68, 69].

The novelty of this study lies in the detection of pathogenic respiratory diseases in chicken flocks, irrespective of numerous factors, such as the type of vaccines used, vaccine programs implemented, levels of biosecurity maintained, geographical locations of the farms, and the intensity of farming. Therefore, this epidemiological study focuses on identifying and understanding the presence of violent respiratory viruses in chicken populations, regardless of preventive measures in place, which may have significant implications for the poultry industry.

## Conclusion

From the results of the present study, it can be concluded that common respiratory virus infections (H5N1, NDV, H9N2, and IBV) are circulating in broiler chickens from January to October 2022 along the Delta region governorate. Our results also revealed that IBV and H9, as single or mixed infections, play a significant role in respiratory infection in broilers. The vaccine is still unable to protect broiler chickens from the development of infection and the transmission of the virus to the poultry environment. Therefore, poultry vaccines need to be regularly evaluated and renovated for face-infective field virus mutants, and poultry farms must also take more biosecurity measures.

The molecular epidemiology of respiratory viral diseases prevalent in Egypt could prompt researchers, veterinarians, and poultry producers to reevaluate existing vaccination strategies, biosecurity protocols, and geographical factors that may contribute to the spread of respiratory diseases among chicken flocks. This can contribute to the development of more effective prevention strategies (vaccine efficacy and vaccination strategies), and the development of effective control measures aimed at mitigating the impact of respiratory diseases on chicken flocks to ensure effective disease management, health protection, food safety, and minimization of economic losses in the poultry industry.

## Authors’ Contributions

AAE, ME, and HMH: Collected samples and conducted a laboratory investigation. AAE, MMA, and ME: Supervised the study and drafted and revised the manuscript. All authors have read, reviewed, and approved the final manuscript.
